# On the usefulness of parental lines GWAS for predicting low heritability traits in tropical maize hybrids

**DOI:** 10.1371/journal.pone.0228724

**Published:** 2020-02-07

**Authors:** Giovanni Galli, Filipe Couto Alves, Júlia Silva Morosini, Roberto Fritsche-Neto

**Affiliations:** 1 University of São Paulo, Luiz de Queiroz College of Agriculture, Department of Genetics, Piracicaba, São Paulo, Brazil; 2 Institute of Quantitative Health Sciences and Engineering, Michigan State University, East Lansing, Michigan, United States of America; INRA France, FRANCE

## Abstract

Genome-wide association studies (GWAS) is one of the most popular methods of studying the genetic control of traits. This methodology has been intensely performed on inbred genotypes to identify causal variants. Nonetheless, the lack of covariance between the phenotype of inbred lines and their offspring in cross-pollinated species (such as maize) raises questions on the applicability of these findings in a hybrid breeding context. To address this topic, we incorporated previously reported parental lines GWAS information into the prediction of a low heritability trait in hybrids. This was done by marker-assisted selection based on significant markers identified in the lines and by genomic prediction having these markers as fixed effects. Additive-dominance GWAS of hybrids, a non-conventional procedure, was also performed for comparison purposes. Our results suggest that incorporating information from parental inbred lines GWAS led to decreases in the predictive ability of hybrids. Correspondingly, inbred lines and hybrids-based GWAS yielded different results. These findings do not invalidate GWAS on inbred lines for selection purposes, but mean that it may not be directly useful for hybrid breeding.

## Introduction

Marker Assisted Selection (MAS; [1]) is a commonly used technique in plant breeding. This methodology has been reported to be most effective for selecting genomic regions that account for a relevant amount of genotypic variation in a population, often identified by QTL mapping. However, many important traits such as grain yield, plant height, and nutrient use efficiency are genetically controlled by many genes, each one accounting for a small percentage of the selectable variation. Therefore, the inability to select for small-effect QTL is an important limitation of MAS [2,3]. In this sense, genomic prediction (GP; [4]), a whole-genome-based selection methodology, was proposed with the intent of capturing as much genetic variation as possible, regardless of QTL identification.

Over the last decades, a realm of GP methods have been suggested for predicting agronomically relevant traits [2]. Bayesian LASSO (Least Absolute Shrinkage and Selection Operator; [5]), BayesA, BayesB [4], RKHS (Reproducing Kernel Hilbert Spaces; [6]), and genomic BLUP [7] are examples of the most notorious. Additionally, further adaptations have been proposed to principal methods such as compressed BLUP, SUPER BLUP [8], and GP with higher-effect markers differentially modeled (e.g., as fixed effects; [3,9,10]).

Simulations and empirical studies have shown that the ability of GP to associate phenotypic patterns to genomic variations is intrinsically related to the genetic architecture of traits [4,11,12]. Hence, the aforementioned GP methods were idealized to match genetic architectures by adjusting *a priori* assumptions regarding the variance and effect of genomic markers. Then, it is inferable that studying the genetic control of traits, in addition to be a crucial breeding practice, may lead to insights regarding the idealization and employment of GP methods.

One of the most popular methods of studying the genetic control is by genome-wide association studies (GWAS). GWAS has been extensively applied to maize populations for important agronomical traits such as foliar architecture [13], seed oil concentration and composition [14], resistance to diseases [15,16], root development [17], nitrogen use related traits [18], and plant height [19]. These studies showed that the methodology is efficient at finding candidate genes from which desirable allelic variants can be selected by breeders [20].

The concept of GWAS is centered at the possibility of using a wide range of populations, with few restrictions regarding the genetic structure of genotypes being utilized. In maize, some GWAS are reported in non-inbred populations (e.g., testcrosses; [21]), but most are carried out on (nearly) inbred genotypes (e.g., inbred association panels; [20]). These studies have shed light on the genetic control of many traits and are available for breeders to use [9]. However, GWAS of lines is only relevant in an cross-pollination breeding sense if important to the development of hybrids.

Hence, markers identified by GWAS can be utilized for MAS or in a MAS + GP combination. Thus, efforts have been employed in the use of GWAS-found markers for whole genomic prediction of the same [10] and different populations (GWAS in population A and prediction of population B; [9]). This approach is efficient in some situations, often increasing prediction ability [9,10]. However, there are still no reports on this methodology in such a way that would fit the conventional maize pipeline, that is, GWAS based on lines performance, but the prediction of hybrids. Furthermore, how relevant are the marker effects estimated when only additive effects are present in a scenario where dominance plays an essential role? This lacking information raises questions on the relevance of parental lines GWAS for hybrid breeding. Therefore, we aimed to evaluate the efficiency of MAS or MAS+GP in a setup focused on maize hybrid breeding, where the GWAS is carried out in inbreed lines, and its results (markers with significant effects) are differentially accommodate in GP models.

## Material and methods

### Phenotype assessment and analysis

To verify the usefulness of inbred line GWAS in hybrid maize breeding, we selected the case study of Low Nitrogen Tolerance Index (LNTI), a critical, lowly heritable trait. Inbred line GWAS on this trait has been reported by Morosini et al. [18]. The genetic material was composed of 906 maize single-crosses [22] obtained in a partial diallel using 49 of the 64 inbred lines studied in Morosini et al. [18] (S1 Table). The 49 inbred lines were selected based on their nitrogen use efficiency [23] and identified as belonging from two heterotic groups, 35 flint and 15 dent [24]. Thorough study of the population of hybrids was previously reported [24,25].

Field trials were carried out in two nitrogen (N) regimes [ideal N (IN; 100 kg ha^-1^) and low N (LN; 30 kg ha^-1^)], at two sites [Piracicaba-São Paulo (22°42'23"S, 47°38'14"W, 535 m) and Anhembi-São Paulo (22°50'51"S, 48°01'06"W, 466 m)], during two years (second growing seasons of 2016 and 2017). The single-crosses (738 in 2016 and 789 in 2017) were phenotyped for grain yield (GY; Mg ha^-1^) which was estimated based on the plot production (7 m row) corrected for 13% moisture. Treatments were arranged in augmented block schemes (unreplicated trials). Each incomplete block was composed of 16 regular treatments and two checks.

A joint model was fit having year, site, nitrogen regime (and interactions) for factor effect significance by Likelihood Ratio Test (LRT; random) and Wald Statistics (fixed). Once evidence of the significance of N application was found, further analyses were performed for each nitrogen level. The adjusted means of single-crosses were estimated for low (GY_LN_) and standard (GY_IN_) N conditions according to the following linear mixed model using ASReml-R [26]:

y=Xβ+Vb+ε

where ***y*** is the yield of each plot; ***X*** is the incidence matrix for check, hybrid, environment (combinations of site and year), and check × environment regarded as fixed with effect ***β***; the random effect of block-within-environment is represented by ***b*** $\left[b∼N(0,I\sigma_{b}^{2})\right]$ with incidence ***V***; ***ε*** $\left[\varepsilon ∼N(0,I\sigma_{\varepsilon }^{2})\right]$ is a vector of residuals based on checks. The adjusted means for GY_LN_ and GY_IN_ were used to estimate the LNTI of the single-cross *i* by ${LNTI}_{i}=(1-{{GY}_{LN}}_{i}/{{GY}_{IN}}_{i})\times 100$ [27].

In a second step, this model was fit considering hybrid, environment, and check × environment as random effects for the estimation of the heritabilities at plot H2=σ^g2/(σ^g2+σ^ga2+σ^ε2) and entry mean level H2=σ^g2/(σ^g2+σ^ga2a+σ^ε2ar) for both N regimes using where σ^g2,σ^ga2, and σ^ε2 are the genotypic (hybrids), genotypic (checks) × environment, and residual variances, respectively. Variances were weighted by the number of environments (*a* = 4) and replications (*r* = 1).

### Genomic data

The forty-nine inbred lines were genotyped with the Affymetrix^®^ Axiom^®^ Array of 614k SNPs [28]. Quality control procedure was applied with: *(1)* removal of low (<95%) call rate markers; *(2)* elimination of loci with at least one heterozygote; *(3)* imputation of the missing data considering only homozygous combinations using the Synbreed-R package [29]; *(4)* prune of markers so the mean linkage disequilibrium (LD) is 0.9 using Plink v. 1.9 algorithms [30]; *(5)* build of the artificial single-crosses genomic matrix combining the genotype of the parents *in silico*; *(6)* exclusion of markers when the minor allele frequency was lower than 5%. Thus, a total of 34,571 markers were kept for subsequent analyses.

Principal Component Analysis (PCA) and Admixture Clustering revealed the existence of two subpopulations in the inbred lines germplasm [18,31]. Additionally, according to Morosini et al. [18], the panel presents a mean length of LD decay of 80–100 kb (*r*^2^ = 0.13). Since no Mendelian sampling occurred from parental lines to single-crosses for the markers considered (homozygous only), the LD decay should remain similar on the offspring. No clear structuration of the population of hybrids was identified through PCA analysis [24]. The distribution of minor allele frequency and heterozygosity on hybrids is reported (S1 Fig). Also, over 99% of markers on hybrids were in Hardy-Weinberg Equilibrium. Additional information on the inbred lines and hybrid population can be found on [24,25].

### Genomic prediction

The genomic matrix (*Z*) was split into two matrices. The markers identified as significant on the parental population for LNTI by Morosini et al. [18] (S2 Table) were allocated in matrix *M*, and the non-significant markers were allocated in matrix *W*.

The predictive ability of the methods was obtained using the training-testing validation scheme with 50 replications. The training population was composed of 75% of the hybrids, randomly assigned. The marker-based model training for prediction of LNTI of the maize hybrids was performed using additive MAS (MAS(A)), dominance MAS (MAS(D)), additive + dominance MAS (MAS(AD)), BayesB, GBLUP, MAS|GBLUP, RKHS, and MAS|RKHS. The MAS|GBLUP, and MAS|RKHS methods are modifications from the original methods to accommodate significant markers identified in GWAS as fixed effects. The methods were applied following the models [1], [4], [6,7]:

g^=Xμ+Mf+e:MAS

g^=Xμ+Zb+e:BayesB

g^=Xμ+Th+e:GBLUPorRKHS

g^=Xμ+Mf+Ts+e:MAS|GBLUPorMAS|RKHS

where g^ is the vector of adjusted phenotypic values of hybrids; ***X*** is the incidence matrix for the mean *μ*; ***M*** is genotype matrix of significant markers with effect ***f*** regarded as fixed; ***Z*** is the whole genomic matrix associated to ***b***; ***T*** is the design matrix for hybrids relating the genetic values ***h*** or ***s*** to the dependent variable. In BayesB, the distribution of the independent vectors was assumed as $b∼\{0\;with\;probability\;\pi ;\;N(0,I\sigma_{\beta }^{2})\;with\;probability\;(1-\pi )\}$ where *π* is the parameter with Beta distribution indicating the proportion of markers with null variance; ***h***~*NID*(0,***G***_*Z*_); ***s***~*NID*(0,***G***_*W*_); The additive relationship matrices were estimated as $G_{Z}=\frac{ZZ^{\prime }}{trace(ZZ^{\prime })/n}$ using all markers and $G_{W}=\frac{WW^{\prime }}{trace(WW^{\prime })/n}$ using only non-significant markers. The Gaussian Kernel is derived from the additive GRMs as in [25]. Models were implemented in the BGLR library [32] of R. MCMC sampling was performed 30,000 times with the elimination of the first 5,000 and thinning of 5. Hyperparameters were applied as presented by Pérez and De Los Campos [28].

The GEBVs of the test population of hybrids (25%) were predicted by GEBV=Mf^ for MAS; GEBV=Zb^ for BayesB; GEBV=Th^ for GBLUP and RKHS; and by GEBV=Mf^+Ts^ for MAS|GBLUP and MAS|RKHS. The training-testing procedure was performed 50 times and the mean *Pearson’s product-moment* correlations of g^ and ***GEBV*** across all iterations represented the predictive ability of the method.

### GWAS

For further comparisons, GWAS was performed on the hybrids, including a dominance effect, considering the single-marker regression following the model:

g^=Xμ+Sm+[Tv+To]+ε

where g^ is the vector of adjusted LNTI, GY_LN,_ or GY_LN_ values of hybrids; ***X*** is the incidence matrix for μ^ which is the vector of fixed effects including mean and population structure (PCA); Number of principal components varied from 0 to 3 and only the ones that led to the best fit were utilized. ***S*** is the additive (aa = -1, Aa = 0 or AA = 1; ***S***_*A*_), dominance (aa = 0, Aa = 1 or AA = 0; ***S***_*D*_) or addivive + dominance (***S***_*A*_***S***_*D*_) genomic incidence matrix associated to fixed marker effect ***m***; ***T*** is the incidence matrix of hybrids related to the vector of polygenic genetic effects with $v∼(0,G_{A}\sigma_{a}^{2})$ and/or $o∼(0,G_{D}\sigma_{d}^{2})$, where $G_{A}=\frac{ZZ^{\prime }}{2\sum_{j=1}^{m}p_{j}(1-p_{j})}$ and $G_{D}=\frac{ZZ^{\prime }}{\sum_{j=1}^{m}{2p}_{j}q_{j}(1-p_{j}q_{j})}$ where *p* and *q* are the allelic frequencies for the j^th^ marker (*j* = 1…*m*) as in [33]; ***ε*** refers to the vector of residuals having $\varepsilon ∼NID(0,{I\sigma }_{\varepsilon }^{2})$. The *p-values* of the markers were tested against a threshold estimated by permutation with 400 repetitions to determine significance. The association analyses were performed using the adapted functions of the Sommer package in R [33]. Additionally, the heritability of significant markers was estimated as H2=2pqα2/Var(g^) for the additive GWAS; for the additive + dominance GWAS, H2=2pq[a+d(q−p)]2/Var(g^) was used for the additive and H2=4p2q2d2/Var(g^) for the dominance effect; where *p* and *q* are the allelic frequencies and *α* is the allelic substitution effect (ASE), *a* is the additive effect and *d* is the dominance deviation of the significant SNP. The regression coefficient obtained by the GWAS regression depends on the type of the modeled effects, and conclusions should be drawn accordingly.

## Results

### Phenotypic analysis

The joint phenotypic analysis of GY revealed significant effects of environment, block within environment, check × environment interaction, and hybrid by LRT test (P<0.05). Additionally, the heritability was 0.25 at plot and 0.57 at entry-mean levels. Under the statistical evidence of nitrogen application effect on yield, the phenotypic analysis was performed in each regime. The heritabilities at the plot level were 0.25 and 0.19 for trials under low and normal nitrogen conditions, respectively. At the entry mean level, it was 0.55 for LN and 0.49 for IN. The adjusted GY values under LN ranged from 3.23 to 8.54 Mg ha^−1^, with mean 6.50 Mg ha^−1^. Under IN the means were higher, varying from 4.24 Mg ha^−1^ to 9.62 Mg ha^−1^, averaging 7.37 Mg ha^−1^. The LNTI, estimated from the mentioned GY means, ranged from -14.54 to 30.78% with mean 11.65% (S2 Fig). Two genotypes were removed based on the LNTI values.

### MAS and genomic prediction

Low magnitude differences between methods regarding genetic and residual variances were found (S3 Table). Nonetheless, RKHS presented the highest genotypic variance values, while GBLUP presented the lowest (for kinship/genetic distance-based methods). The residual variance was greater when GBLUP or MAS|GBLUP was utilized and lowest for RKHS. In addition, it is clear that when GP and MAS are combined, genetic/genotypic variation decreases. Conversely, the residual variance did not present a specific performance pattern regarding the combination of methodologies. MAS had no genetic variance as residual was the only random component. Furthermore, all presented the same residual variance, which was higher than the genome-wide based methods.

**Fig 1:**
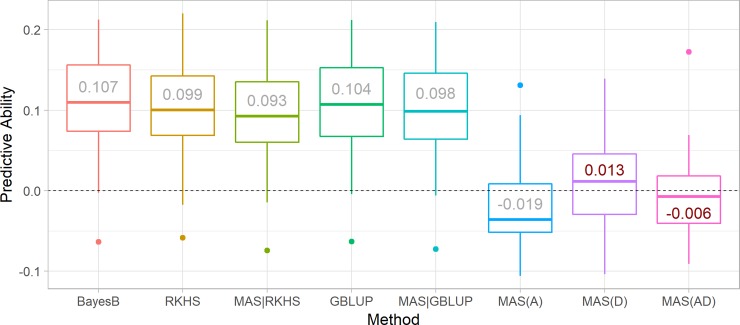
Predictive ability of LNTI in maize single-crosses using BayesB, RKHS, MAS|RKHS, GBLUP, MAS|GBLUP, additive MAS, dominance MAS, and additive + dominance MAS. The MAS is based on four markers identified as significantly associated with the trait in the parental inbred lines by Morosini et al. [18]. Values inside the boxes are mean correlations across 50 replicates. Red font indicates that the mean is not statistically different from zero by *t-test*.

Low prediction ability (PA) values for LNTI were found (Fig 1). Values ranged from 0.093 to 0.107 under GP methods, obtained using MAS|RKHS and BayesB, respectively. From the MAS methods, MAS(D) presented the higher PA (0.013), while MAS(A) yielded the lowest (-0.019). Regardless of the model, PAs of MAS(D) and MAS(AD) were not significantly divergent from zero by *t-test*. Furthermore, the results indicate that integrating GP and MAS (MAS|GBLUP and MAS|RKHS) under the studied scenario did not lead to any prediction improvements.

### Genome-wide association analysis based on hybrids performance

**Fig 2:**
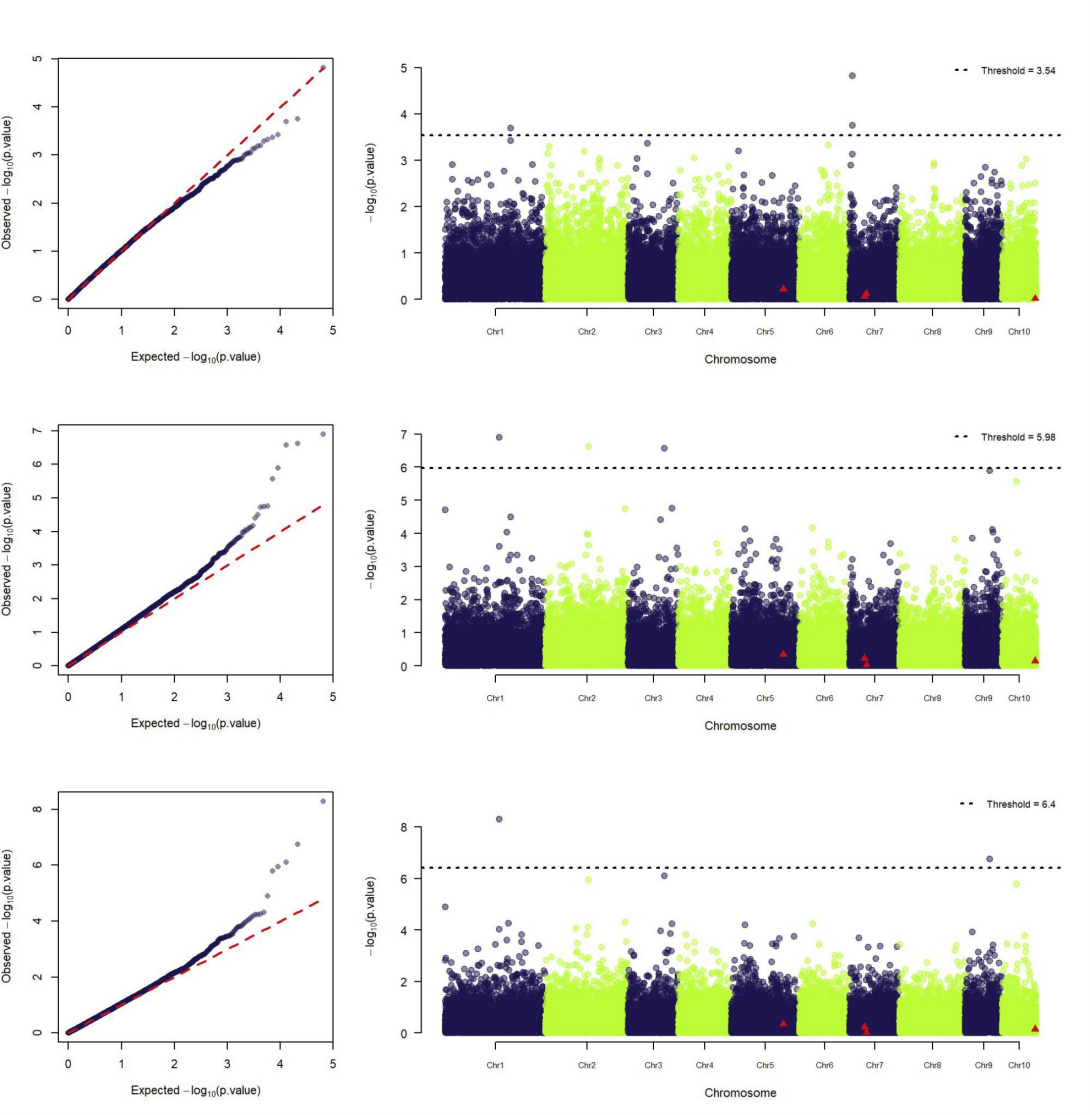
Manhattan and QQ-plots of GWAS for low nitrogen tolerance index (LNTI) of 904 maize single-crosses using 34,571 markers. Plots represent additive effect with model *S*_*A*_+*G*_*A*_ (top); *S*_*AD*_+*G*_*A*_ (middle); and dominance with model *S*_*AD*_+*G*_*D*_ (bottom). Significant markers identified in the inbred lines by Morosini et al. [18] are represented by red triangles in the Manhattan plot.

GWAS was carried out under a series of model setups regarding the control of polygenic effects using different relationship matrices (*G*_*A*_, *G*_*D*_ or *G*_*AD*_) and the number of principal components for controlling population structure (0, 1, 2, and 3). Using a single principal component led to the best adjustments, and only analyses with the best QQ plots were reported (Fig 2). Eight marker-trait associations were found for LNTI for the additive and dominance effects. One of the markers was significant for both additive and dominance effects, totalizing then seven exclusive markers (Table 1). Chromosomes 1 and 7 had two significant markers, and 2, 3, and 9 had one. However, the two markers identified at chromosome 7 are fairly close (LD of 0.83) and probably tracking the same genomic variant.

**Table 1:** List of markers significantly associated with LNTI, GYLN, and GYIN by GWAS of 904 hybrids with 34,571 markers with chromosome (Chr), position (in base pairs), minor allele frequency (MAF), type of effect tested, value of the marker regression coefficient (*β*), marker effect heritability (H^2^), gene identification (Gene ID), distance between gene and marker, and annotation.

Trait	#	Marker	Chr*	Position (bp)	MAF	Effect	*β*	H^2^	Gene ID	Distance from Marker (bp)ª	Annotation
LNTI	1	Affx.90980373	1	170,850,946	0.08	A/D**	-9.56/10.60	0.0017/0.0645	-	-	-
	2	Affx.91015157	1	223,625,482	0.25	A	-1.60	0.0254	Zm00001d032353/LOC100281086	-48,051	phot2—blue-light receptor phototropin 2
									Zm00001d032356/LOC103643337	-14,446	putative wall-associated receptor kinase-like 16
	3	Affx.90589560	2	162,469,457	0.08	A	-9.39	0.0151	Zm00001d005176/LOC103647207	-36,575	Protein kinase-like domain
									Zm00001d005177/ LOC100276832	-22,015	maternal effect embryo arrest 60
	4	Affx.90263516	3	210,763,644	0.06	A	-11.38	0.0094	Zm00001d043808/ LOC100275175	-17,061	DUF1639 family protein
									Zm00001d043809/LOC100274030	-76	Uncharacterized
									Zm00001d043812/LOC103651327	14,025	ipt3B - isopentenyl transferase3B adenylate isopentenyltransferase
	5	Affx.90918032	7	7,456,904	0.10	A	-2.30	0.0252	Zm00001d018855/LOC103631967	-2,775	putative carboxylesterase 15
									Zm00001d018856/LOC103631968/LOC103633695	2,505	cold-regulated 413 plasma membrane protein 1-like 14-3-3-like protein GF14 nu
									Zm00001d018857/LOC103631970	44,889	probable carboxylesterase 15
	6	Affx.90609217	7	7,456,919	0.15	A	-2.32	0.0364	^SAP^	^SAP^	^SAP^
	7	Affx.91242936	9	133,625,758	0.08	D	9.50	0.0518	Zm00001d047525/LOC103633718	-53,024	Heat shock protein 70 family luminal-binding protein 3-like
									Zm00001d047526/ LOC100273998	3,292	Lung seven transmembrane receptor family protein
GY_LN/IN_	8	Affx.90227892	3	180,660,986	0.05	A	-1.48/-1.69	0.0018/0.0050	-	-	-
GY_IN_	9	Affx.91283875	5	58,138,755	0.05	D	0.69	0.0099	Zm00001d014665/LOC100274487	19,925	Subtilisin-like protease SBT2.6
									Zm00001d014666/LOC100272702	27,245	Pentatricopeptide repeat-containing
									Zm00001d014667	32,416	DUF4228 domain protein
									Zm00001d014668	35,507	Core-2/I-branching beta-16-Nacetylglucosaminyltransferase family protein

^SAP^ same as previous

*Chromosome

**A: additive; D: dominance

ªConsidering the closest gene position to the marker. Positive and negative values: genes are upstream and downstream the marker, respectively

The comparison of physical locations of significant markers by GWAS with the B73 reference genome allowed the identification of several candidate genes. Putative genes were found on chromosome 1 (phot2—blue-light receptor phototropin 2; putative wall-associated receptor kinase-like 16), 2 (Protein kinase-like domain; maternal effect embryo arrest 60), 3 (DUF1639 family protein; ipt3B - isopentenyl transferase3B adenylate isopentenyltransferase), 7 (putative carboxylesterase 15; cold-regulated 413 plasma membrane protein 1-like 14-3-3-like protein GF14 nu; probable carboxylesterase 15), and 9 (Heat shock protein 70 family luminal-binding protein 3-like; Lung seven transmembrane receptor family protein).

**Fig 3:**
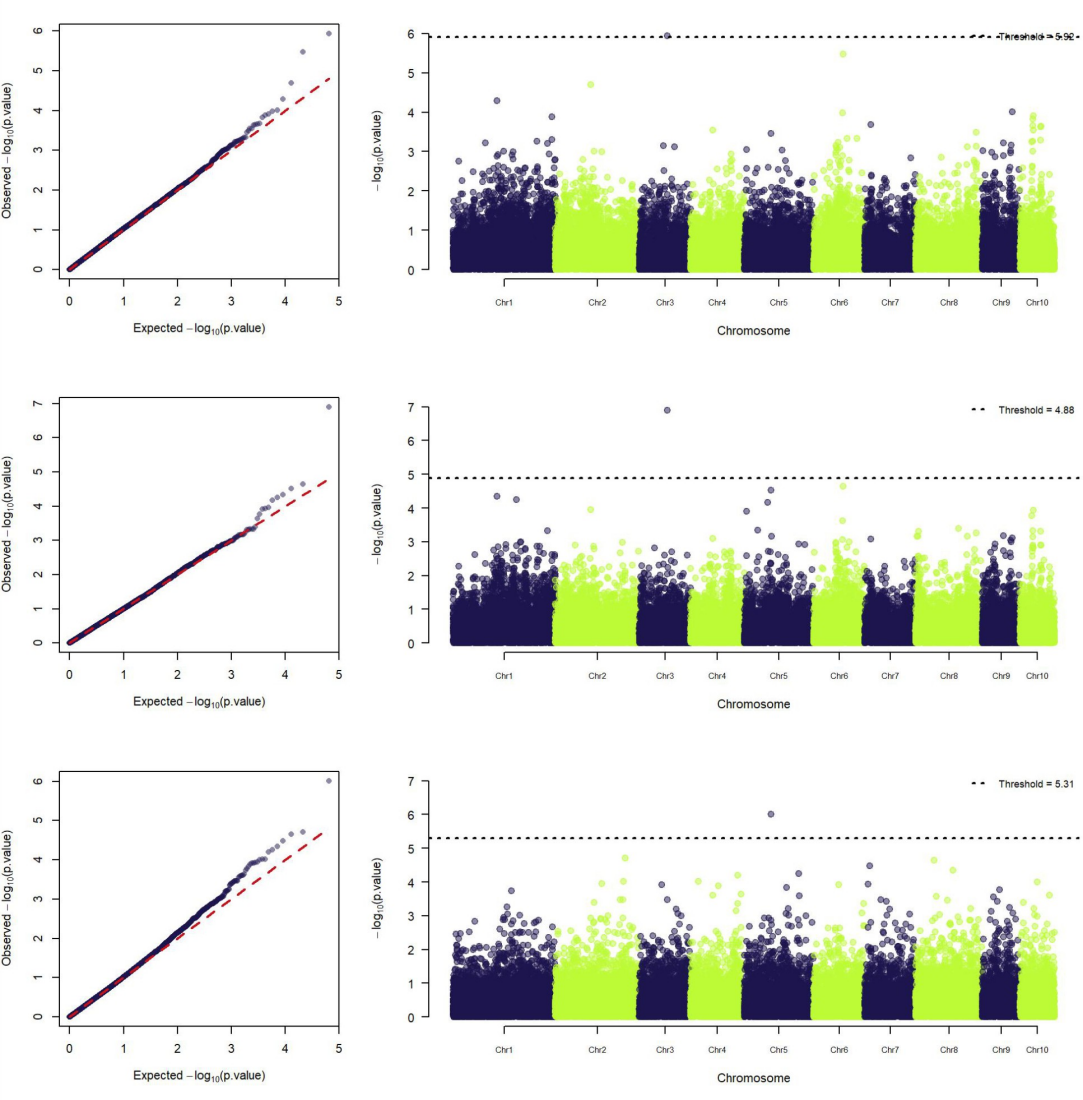
**Manhattan and QQ-plots of GWAS for GY**_**LN**_ **(Top) and GY**_**IN**_ **(middle and bottom).** Plots represent additive effect with model *S*_*AD*_+*G*_*AD*_ (top and middle); and dominance effect with model *S*_*D*_+*G*_*AD*_ (bottom). Analyses were carried out on 904 maize single-crosses using 34,571 markers.

**Fig 4:**
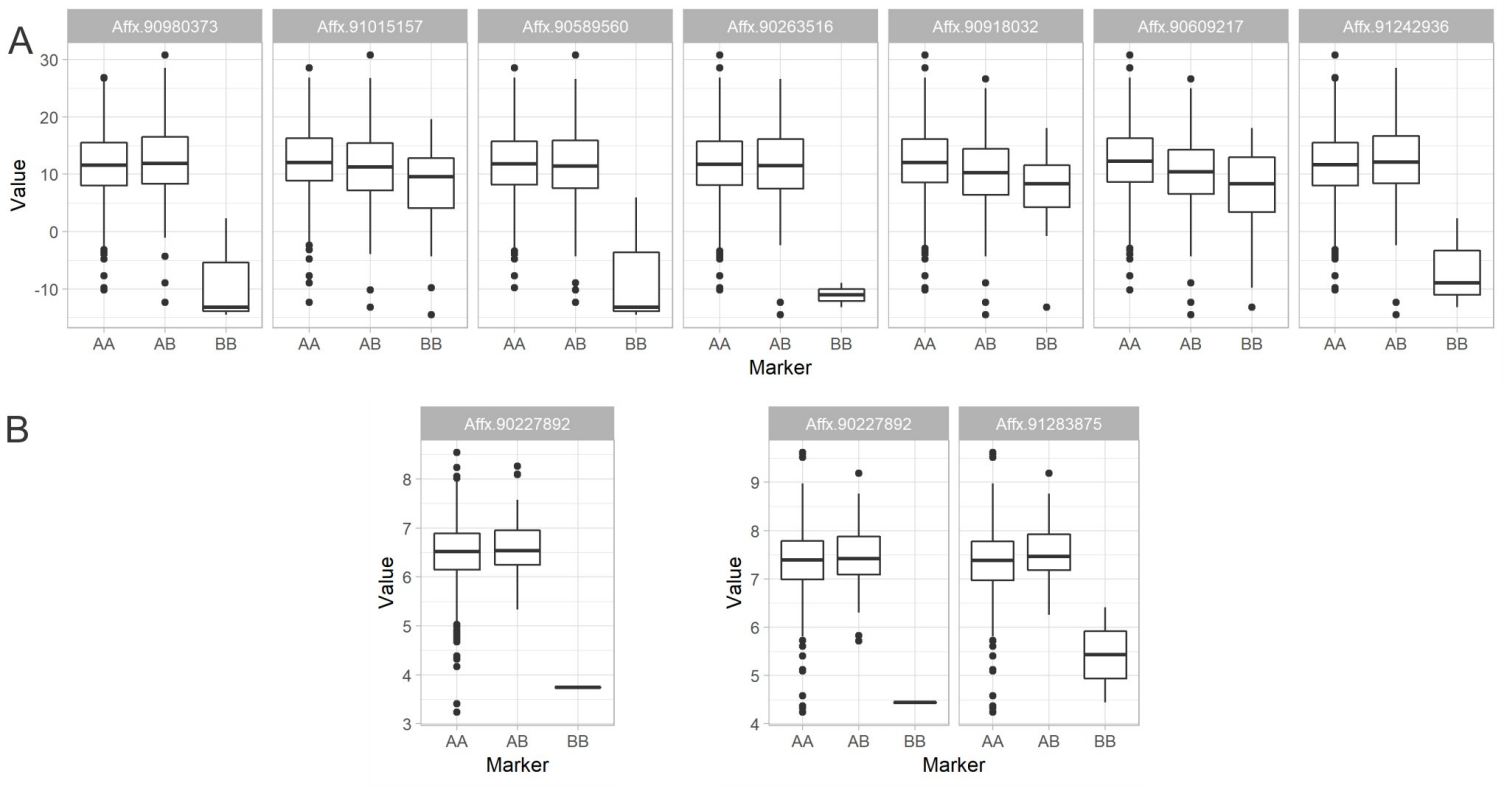
**A) Boxplot of LNTI adjusted means (%) by genotypic classes for significant markers.** Affx.90980373 was significant for both additive and dominance effects. Affx.91242936 was significant for dominance effect. All other markers were significant for the additive effect; **B) Boxplot of GY**_**LN**_ **(left) and GY**_**IN**_ **(right) adjusted means (Mg ha**^**-1**^**) by genotypic classes for significant markers.** Affx.91283875 was significant for dominance effect. Affx.90227892 was significant for additive effect.

Additionally, GWAS for GY_LN_ and GY_IN_ were also reported (Figs 3 and 4B) using the same model criteria as for LNTI (best QQ plot). Three associations were found with GY, being two with ideal N and one with low N. The marker identified on both regimes was located on the chromosome 3 and was of additive effect. The marker identified solely under standard N is on chromosome 5 and was found with the dominance model. When compared to the reference genome, this SNP had four putative genes on its range (Subtilisin-like protease SBT2.6; Pentatricopeptide repeat-containing; DUF4228 domain protein; Core-2/I-branching beta-16-Nacetylglucosaminyltransferase family protein). However, for the marker identified for both GY_LN_ and GY_IN_ on chromosome 3, no trace was found to any gene.

Absolute values of regression coefficient ranged from 1.6 to 11.38 for LNTI. For GY, values were from 0.69 to 1.69. The heritability of the markers (H^2^) varied from 0.0017 to 0.0645 for LNTI and from 0.0018 to 0.0099 for GY. It is essential to notice that some regression coefficients could be overestimated.

## Discussion

### On the combination of MAS and GP

Bernardo [3] introduced the concept of combining MAS and GP. Ever since, reports have validated the methodology [9–11]. Our results showed no advantage in combining MAS and GP for predicting LNTI, a low-heritability highly polygenic trait [18], of tropical maize hybrids. That corroborates the findings of Bernardo [3], Li et al. [34], and Spindel et al. [10], which suggest that differentially modeling significant markers improve prediction performance only when the trait is highly heritable and the markers explain a fair proportion of the genetic variance. In our case, none of these prerequisites were met. Additionally, modeling major markers as fixed may create a confounding effect between genetic fixed and random terms which is dealt with when all markers are random. Regarding the prediction method, no much difference was found between the GP and MAS + GP methods, meaning that the more straightforward method (namely GBLUP) can be utilized with no predictive ability loss. Additionally, the inferior (and non-statistically divergent to zero) predictive ability of MAS makes it a poor candidate method for prediction.

Our results suggest that there is no advantage in modeling markers identified in parental lines as fixed effects for the prediction of hybrids in both MAS and MAS + GP scenarios. It might be due to the lack of connection between the genotype-phenotype relationship of parental inbred lines and hybrids given the changes in the allele substitution effect. Allele substitution effect (*α*) of a given gene depends on the allele frequencies (*p* and *q*) and genotypic values (*a* and *d*), since *α* = *a*+*d*(*q*−*p*) [35]. Despite being derived from a partial diallel, marker allelic frequencies of parental lines and hybrids were fairly similar (S2 Table). Regarding genotypic values, *α* of any marker in inbred lines would be represented by *α* = *a*, since *d* = 0. On the other hand, the phenotype of hybrids is believed to be consistently driven by dominance effects (high *d*) due to the expression of high-level heterosis in the species.

After all, predicting traits based on information extracted from the parental inbred lines *per se* information has been reported not to be effective for polygenic traits due to the masking of non-additive effects and genotype by environment interactions [36–38]. By applying MAS with the markers identified by Morosini et al. [18], our results showed that the covariance between the hybrids genetic values predicted from marker effects estimated from parental lines and hybrids was negative (S3 Fig). This reinforces the inability to predict performance of hybrids based on parental information. Besides, the significant markers identified in hybrids and parental lines were not the same (Fig 2; [18]). That means that not only the difference in nature of effects (additive and dominance), but also genomic regulation toward potentially distinct regions/loci (genes expressed in heterozygous forms) are relevant for the differential establishment of lines and hybrids phenotypes. Hence, in a prediction-based breeding context, the results suggest that parental lines-based GWAS might not be meaningful for hybrid breeding for such traits as LNTI.

Although there is strong evidence that carrying out GWAS on hybrids seems a better fit in a breeding context, some factors may hamper the direct use of GWAS in the conventional pipeline. First, it conceptually requires diverse germplasm for maximizing the quality of results. It might not be achieved in breeding populations, which commonly fixed for the majority of favorable alleles (elite germplasm). In Fig 4, the genotypes are ordered by frequency, indicating that for the significant markers, favorable alleles were in higher frequency. Secondly, finding functional associations that can be used to implement genetic gain is sometimes occasional due to the genetic architecture of the trait. These factors should be taken into account by the breeder in the decision-making process.

Simulations have shown which scenarios are favorable for utilizing GWAS-significant markers/major genes in GP [3,11]. Our work aggregates new insights as putative QTL are identified in parents (inbred lines) and GP is applied to hybrids. The results showed no advantage when combining GWAS, and GP in this framework. Nevertheless, further studies on traits with different genetics architecture are still to be carried out.

### Additive + dominance GWAS of maize hybrids

Modeling dominance effect in GP of maize hybrids is a recurrent practice that generally yields favorable results [25,39]. However, GWAS have been mostly limited to the identification of allelic substitution effects, even when non-inbred populations were evaluated [21]. Some reports on human [40], beef-cattle [41], sunflower [42], and pig populations [43] revealed the ability of additive-dominance-based GWAS to identify functional polymorphisms and lead to promising candidate genes. In the current study, RKHS, a non-linear GP method, did not outperform the additive-based models. Nevertheless, two out of seven significant markers identified for LNTI were based on the dominance parametrization. For GY, under standard nitrogen regime, one out of two was based on dominance. This evidence manifests the importance of assessing non-additive effects for finding functional polymorphisms in hybrids.

GWAS suggested eleven significant marker-trait associations, eight for LNTI and three for GY under two N fertilization regimes. The annotations revealed that seven out of nine markers were flanked by putative genes. However, some associations presented unrealistic (overestimated) regression coefficients (*β*; allele substitution effects/additive effect/dominance deviations). These markers had in common a low MAF (<0.10), which might be leading to these results as well as other factors such as the Beavis effect [44]. Relating effect and genotype frequencies for LNTI, four markers (Affx.90980373, Affx.90589560, Affx.90263516, Affx.91242936) presented a complete dominance-like behavior (Fig 4A). However, only two of those were found significant in the dominance GWAS (Affx.90980373 and Affx.91242936). This pattern seems to be generated by chance in the set of low MAF markers. For GY, one marker was significant in the dominance GWAS (Affx.91283875), but all markers presented the dominance-like pattern, which is also probably driven by their low MAF. However, these aspects do not invalidate the findings but suggest that the use of this information should be done with further attention.

The markers with MAF>0.10, Affx.91015157 and Affx.90918032 (or Affx.90609217), presented the lowest regression coefficients for LNTI, which are consistent with the expected genetic architecture of the trait. Additionally, H^2^ values were relatively low for all markers which is in accordance with the low heritability of LNTI (~0.13 for inbred lines, [18]). This suggests that, even when carried out in hybrids, GWAS findings might be of limited use for selection purposes. Nevertheless, results showed associations to other markers than those from parental lines (Fig 2; [18]).

The putative genes found in the inbred lines were associated with transcription regulation, DNA repair, lipid biosynthesis, and GMP Synthetase. There was no direct correspondence among the putative genes found in hybrids compared to the ones identified in the lines panel [18]. Nevertheless, the physiological implications of the nitrogen are hardly straightly connected to specific genes or functions, given their complex nature. GY under different N regimes and the LNTI are useful tools to assess the N use efficiency (NUE), an important aspect and also a complex trait for maize breeding. In this context, indirect inferences on the role of N are useful towards a better understanding of its genetic architecture.

Regarding the GWAS in maize hybrids, the putative genes are mostly related to the expression of chloroplast genes; cell elongation and plant development; catalytic function of protein kinases, which operates as an on/off switch for many cellular processes, including physiological ones; cellular response to heat, and cytokinin biosynthesis. Therefore, due to the traits evaluated, all these processes are very likely related to the amount of nitrogen available to the plant.

### Implications on cross-pollination breeding for low heritability traits

The GWAS results suggest the presence of both additive and dominance effects controlling LNTI in maize hybrids (Table 1). The breeding scheme for this type of variety is based on heterotic groups combined by diallel mattings and/or testers. In this sense, if the associations are considered of value (e.g., high ASE or H^2^), LNTI can be improved by increasing the frequency of favorable alleles of additive genes in all groups or; increasing the frequency of each allele in the respective groups for genes expressing dominance effects. Morosini et al. [18] reported that that many genes of small effect control LNTI. We added to this finding further functional polymorphisms that control the trait (Fig 2, Table 1). The aspects of genetic architecture, the high number of alleles, and low heritability [18,31] make it hard to predict and select superior genotypes.

After all, the purpose of this work is not convincing that GWAS should only be performed in hybrids. Regardless, our results show that, under situations similar to the one here exposed, GWAS performed in hybrids and lines lead to contrasting results, meaning that inbred-line GWAS might not be directly used for breeding. Nevertheless, it is still relevant as a tool for understanding the physiological base of traits and should not be neglected.

## Supporting information

S1 Fig The distribution of minor allele frequency (left) and heterozygosity (right) on the utilized population of maize hybrids.
(DOCX)

S2 Fig Density plot of grain yield under two nitrogen application regimes (top) and low nitrogen tolerance index (LNTI) of 904 maize hybrids (bottom).
(DOCX)

S3 Fig Variances and covariances of MAS predicted values with four markers identified as significantly associated with low nitrogen tolerance index (LNTI) in maize lines by Morosini et al. (2017). (DOCX)

S1 Table Number of times each line was used as genitor for obtaining the 904 hybrids analyzed. (DOCX)

S2 Table Attributes of four markers on 64 inbred lines and 904 single-crosses (offspring). (DOCX)

S3 TableGenetic ($\sigma_{a}^{2}$) and residual ($\sigma_{e}^{2}$) variances from genomic prediction validation of LNTI in maize single-crosses using BayesB, RKHS, MASS|RKHS, GBLUP, MAS|GBLUP, additive MAS (MAS(A)), dominance MAS (MAS(D)) and additive + dominance MAS (MASS(AD)).(DOCX)
