# Supplementary material for: On the usefulness of parental lines GWAS for predicting low heritability traits in tropical maize hybrids
Source: PLoS One. 2020 Feb 7;15(2):e0228724. doi: 10.1371/journal.pone.0228724 (PMC7006934; doi:10.1371/journal.pone.0228724)
Supplement: S1 Fig — The distribution of minor allele frequency (left) and heterozygosity (right) on the utilized population of maize hybrids. (DOCX) [file pone.0228724.s001.docx]

**
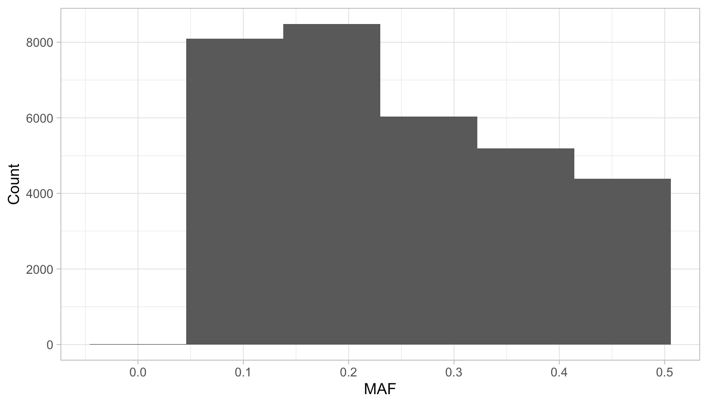

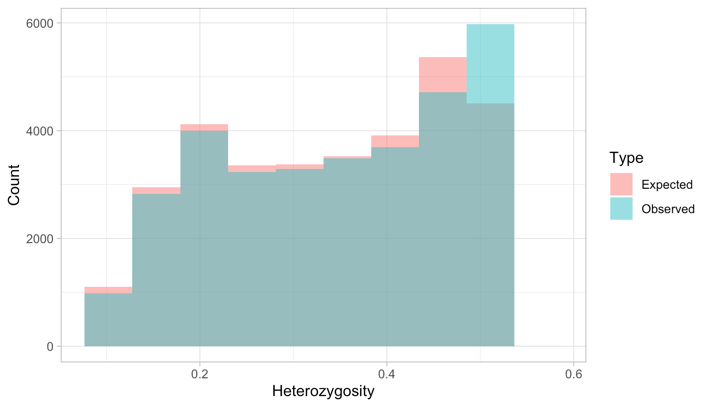
**

**S1 Fig. The distribution of minor allele frequency (left) and heterozygosity (right) on the utilized population of maize hybrids.**
