# Supplementary material for: On the usefulness of parental lines GWAS for predicting low heritability traits in tropical maize hybrids
Source: PLoS One. 2020 Feb 7;15(2):e0228724. doi: 10.1371/journal.pone.0228724 (PMC7006934; doi:10.1371/journal.pone.0228724)
Supplement: S2 Fig — Density plot of grain yield under two nitrogen application regimes (top) and low nitrogen tolerance index (LNTI) of 904 maize hybrids (bottom). (DOCX) [file pone.0228724.s002.docx]

**
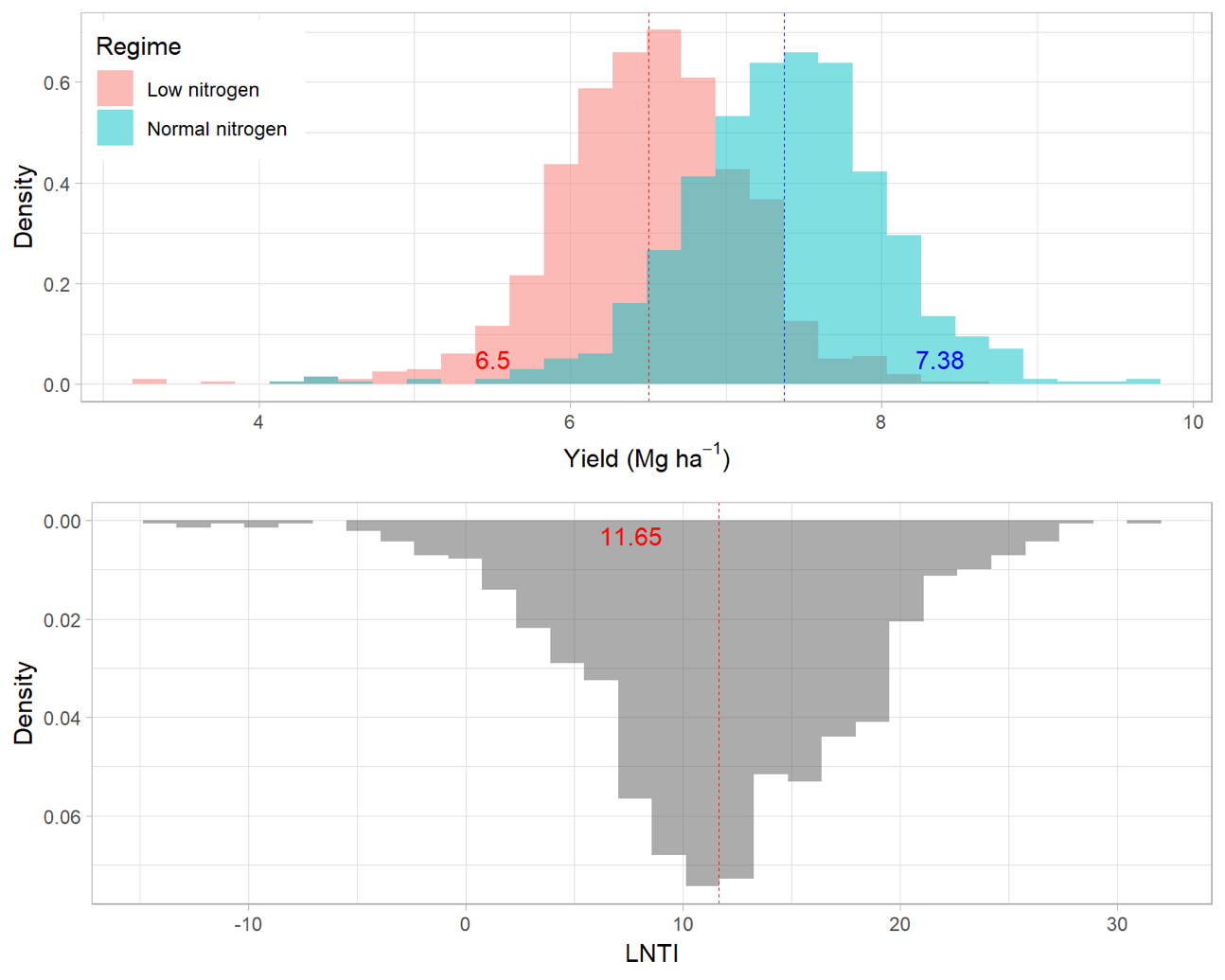
**

**S2 Fig. Density plot of grain yield under two nitrogen application regimes (top) and low nitrogen tolerance index (LNTI) of 904 maize hybrids (bottom).**
