# Supplementary material for: On the usefulness of parental lines GWAS for predicting low heritability traits in tropical maize hybrids
Source: PLoS One. 2020 Feb 7;15(2):e0228724. doi: 10.1371/journal.pone.0228724 (PMC7006934; doi:10.1371/journal.pone.0228724)
Supplement: S3 Fig — (DOCX) [file pone.0228724.s003.docx]

**
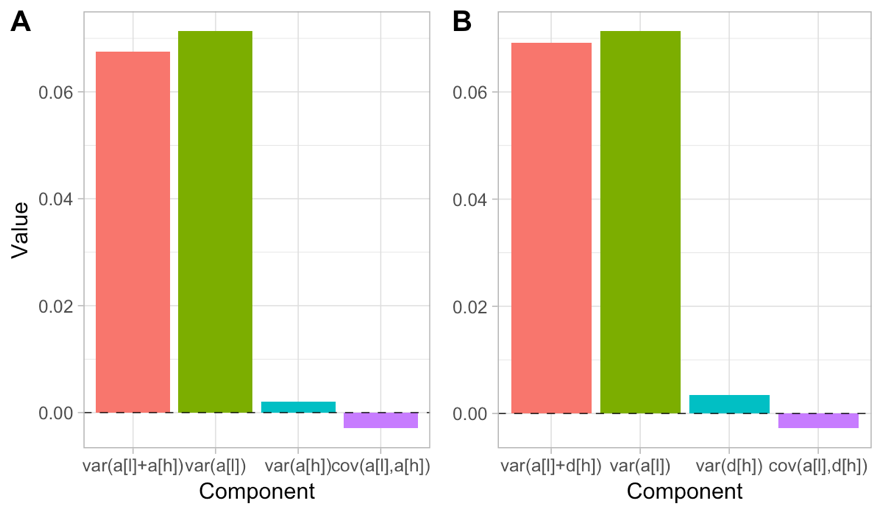
**

**S3 Fig.** **Variances and covariances of MAS predicted values with four markers identified as significantly associated with low nitrogen tolerance index (LNTI) in maize lines by Morosini et al. (2017).** The term var(a[l]) represents the variance of genomic breeding values of hybrids with marker effect estimated on lines; var(a[h]) represents the variance of genomic breeding values of hybrids with marker effect estimated on hybrids; var(d[h]) represents the variance of genomic genotypic values of hybrids with marker effect estimated on hybrids. A) var(a[l]+a[h]) and B) var(a[l]+d[h]) are the variance of the sum of genomic breeding values/genotypic values of hybrids with marker effects estimated on lines and hybrids. cov(a[l],a[h]) and cov(a[l],d[h]) are the covariances of the sum of genomic breeding values/genotypic values of hybrids with marker effects estimated on lines and hybrids, estimated by difference.
