# Supplementary material for: On the usefulness of parental lines GWAS for predicting low heritability traits in tropical maize hybrids
Source: PLoS One. 2020 Feb 7;15(2):e0228724. doi: 10.1371/journal.pone.0228724 (PMC7006934; doi:10.1371/journal.pone.0228724)
Supplement: S1 Table — (DOCX) [file pone.0228724.s004.docx]

**S1 Table**. **Number of times each line was used as genitor for obtaining the 904 hybrids analyzed.**

| **Line** | **#** | **Line** | **#** | **Line** | **#** | **Line** | **#** | **Line** | **#** |
| --- | --- | --- | --- | --- | --- | --- | --- | --- | --- |
| L001 | 20 | L012 | 30 | L022 | 18 | L033 | 40 | L045 | 42 |
| L002 | 34 | L013 | 37 | L023 | 45 | L034 | 43 | L046 | 48 |
| L003 | 44 | L014 | 33 | L024 | 38 | L035 | 40 | L047 | 40 |
| L005 | 32 | L014B | 31 | L025 | 31 | L037 | 40 | L048 | 43 |
| L006 | 41 | L014C | 37 | L026 | 39 | L038 | 39 | L049 | 44 |
| L007 | 33 | L015 | 46 | L027 | 31 | L039 | 38 | L054 | 20 |
| L007B | 34 | L016 | 39 | L028 | 27 | L041 | 38 | L055 | 45 |
| L008 | 43 | L017 | 35 | L029 | 43 | L042 | 35 | L056 | 41 |
| L010 | 42 | L018 | 36 | L031 | 34 | L043 | 40 | L058 | 33 |
| L011 | 41 | L019 | 29 | L032 | 41 | L044 | 35 | - | - |

# Number of times the line was used as genitor
