# Supplementary material for: On the usefulness of parental lines GWAS for predicting low heritability traits in tropical maize hybrids
Source: PLoS One. 2020 Feb 7;15(2):e0228724. doi: 10.1371/journal.pone.0228724 (PMC7006934; doi:10.1371/journal.pone.0228724)
Supplement: S2 Table — (DOCX) [file pone.0228724.s005.docx]

**S2 Table.** **Attributes of four markers on 64 inbred lines and 904 single-crosses (offspring).** The presented markers were identified as significantly associated with the referred trait by Morosini et al. (2017), where only the additive model was used to perform the GWAS.

| **Marker** | **Chromosome** | **Position (bp)** | **q** | |
| --- | --- | --- | --- | --- |
|  |  |  | **Lines** | **Hybrids** |
| Affx.91269353 | 10 | 148,504,575 | 0.32 | 0.35 |
| Affx.90760172 | 7 | 85,581,217 | 0.32 | 0.27 |
| Affx.90464448 | 7 | 98,855,426 | 0.22 | 0.27 |
| Affx.91405933 | 5 | 200,437,599 | 0.21 | 0.18 |

Adapted from Morosini et al. (2017)

^q^ Frequency of the minor allele
