# Supplementary material for: On the usefulness of parental lines GWAS for predicting low heritability traits in tropical maize hybrids
Source: PLoS One. 2020 Feb 7;15(2):e0228724. doi: 10.1371/journal.pone.0228724 (PMC7006934; doi:10.1371/journal.pone.0228724)
Supplement: S3 Table — (DOCX) [file pone.0228724.s006.docx]

**S3 Table. Genetic (**$\boldsymbol{\sigma}_{\boldsymbol{a}}^{\boldsymbol{2}}$**) and residual (**$\boldsymbol{\sigma}_{\boldsymbol{e}}^{\boldsymbol{2}}$**) variances from genomic prediction validation of LNTI in maize single-crosses using BayesB, RKHS, MASS|RKHS, GBLUP, MAS|GBLUP, additive MAS (MAS(A)), dominance MAS (MAS(D)) and additive + dominance MAS (MASS(AD)).** The MAS is based on four markers identified as significantly associated to the trait in the parental inbred lines by Morosini et al. (2017).

| Method | $\boldsymbol{\sigma}_{\boldsymbol{a}}^{\boldsymbol{2}}$ (sd) | | $\boldsymbol{\sigma}_{\boldsymbol{e}}^{\boldsymbol{2}}$ (sd) | |
| --- | --- | --- | --- | --- |
| GBLUP | 0.15 | (0.04) | 0.96 | (0.05) |
| MAS\|GBLUP | 0.11 | (0.03) | 0.96 | (0.05) |
| RKHS | 0.28 | (0.07) | 0.92 | (0.05) |
| MAS\|RKHS | 0.21 | (0.06) | 0.94 | (0.05) |
| BayesB* | 0.03 | (0.01) | 0.85 | (0.01) |
| MAS(A)* | - | | 1.00 | (0.05) |
| MAS(AD)* | - | | 1.00 | (0.05) |
| MAS(D)* | - | | 1.00 | (0.05) |

*Method based on markers as covariates
